# Adoption of Biologically Effective Dose of the Non-Target Lung Volume to Predict Symptomatic Radiation Pneumonitis After Stereotactic Body Radiation Therapy With Variable Fractionations for Lung Cancer

**DOI:** 10.3389/fonc.2020.01153

**Published:** 2020-07-31

**Authors:** Yuxin Jiao, Yanping Ren, Weiqiang Ge, Libo Zhang, Xiangpeng Zheng

**Affiliations:** Department of Radiation Oncology, Huadong Hospital, Fudan University, Shanghai, China

**Keywords:** lung cancer, stereotactic body radiation therapy (SBRT), radiation pneumonitis, biological effective dose (BED), risk factors

## Abstract

**Background:** This study aims to establish lung biologically effective dose (BED)–based uniform dosimetric constraints for minimizing the risk of symptomatic radiation pneumonitis (SRP) from stereotactic body radiation therapy (SBRT) using variable fractionations in patients with lung tumors.

**Materials and Methods:** A total of 102 patients with primary or oligometastatic lung tumors treated with SBRT in our institution were enrolled into this study. The associations between the clinical and dosimetric parameters and the incidences of SRP were analyzed using univariate and multivariate Cox regression hazard models. The receiver operating characteristic (ROC) curve was generated to evaluate the predictive performance of lung BED on the SRP risk compared with the physical dose.

**Results:** SRP occurred in 11 patients (10.8%). In univariate analysis, the mean lung dose (*p* = 0.002), V_5_ (*p* = 0.005), V_20_ (*p* < 0.001), and the percentage of non-target normal lung volume receiving more than a BED of 5–170 Gy (V_BED5−170_, *p* < 0.05) were associated with SRP. Multivariate logistic regression analysis showed that there existed a significant statistical correlation between SRP and V_BED70_ (*p* < 0.001), which performed better than V_5_ or V_20_ on the ROC curves, resulting in an optimal cut-off value of lung V_BED70_ of 2.22%.

**Conclusions:** This retrospective study indicated that non-target lung BED may better predict SRP from patients with SBRT-treated lung cancer. Limiting the lung V_BED70_ below 2.22% may be favorable to reduce the incidence of SRP, which warranted further prospective validation.

## Introduction

Surgical resection still remains the standard of care for patients with operable early-stage non-small cell lung cancer (NSCLC). For those patients with inoperable conditions, the hypofractionated stereotactic body radiation therapy (SBRT) can achieve better outcomes than conventional radiation therapy, and even comparable efficacy with surgery in terms of local control and overall survival (1, 2). Of note, the application of SBRT has been expanding to oligometastatic intrapulmonary (and extrapulmonary) lesions from any solid tumors (3).

Despite the favorable safety profile, various SBRT-related adverse effects have been reported with the predominance of radiation pneumonitis (RP). Mounting evidence has shown that the incidence rate of RP could be higher than 50%, and the percentage of symptomatic RP (SRP, grade ≥2 RP) ranges from 9 to 28% (4). Considering the fragile conditions frequently present in SBRT-treated patients, SRP could compromise the quality of life of patients and subsequently increase hospital admission as well as mortality rate (5–7). Hence, special attentions and endeavors should be invested to minimize the incidence of SRP during the initial evaluation and radiotherapy treatment planning.

Multiple retrospective studies have reported associations between the probability of SRP and dosimetric parameters, including ipsilateral or bilateral mean lung dose (MLD), V2.5–50, planning target volume (PTV) volume or maximal dose, and internal target volume (ITV) (4, 8, 9). However, the clinical applicability of dosimetric constraints has been compromised due to conflicting or inconsistent results mainly attributed to variable fractionation regimens. Several guidelines or consensuses have been published on organ at risk (OAR) dose constraints in specific lung SBRT regimens (3, 5, or 8 fractions) (10). Still, the complexity of clinical scenario exceeds the guidelines, reflected by more dose regimes (from 1 to 10 fractions) and varying biologically effective dose (BED) to tumors.

BED has been proven to be a reliable uniform dosimetric index for understanding tumor and normal tissue response to various fractionation schemes, especially in SBRT. A BED above 100 Gy to the tumor has been reported to be associated with better local control and overall survival in SBRT-treated early-stage NSCLC and widely accepted as the standard dose prescription. However, due to the variation in fractionation schemes, guidelines or consensus on dose constraints of OARs are only fractionation dependent. For example, dose limits of bronchus in commonly used 3-, 5-, or 8-fraction SBRT has been available but lacking in other SBRT or SBRT-like schemes (up to 10 fractions). Also, even for those available dose constraints, inconsistency exists among institutions and in the literature, which together causes difficulties in evaluation and comparison of treatment-related toxicities, especially SRP. Hence, this retrospective study aimed to establish lung BED–based uniform dosimetric constraints for minimizing the risk of SRP from SBRT using variable fractionations in patients with primary or oligometastatic lung tumors.

## Materials and Methods

### Patient Enrollment

Patients with primary or metastatic malignant lung tumors receiving SBRT treatment in our department from October 2011 through June 2019 were enrolled into this study. Their medical records and radiation treatment planning data were retrieved from the database for analysis.

Before SBRT treatment, pathologic confirmation of NSCLC or metastatic lung tumors was required unless patients declined to or were not physically suitable for invasive pathological procedures. In the latter case, clinical diagnosis was made based on the growing patterns of persistent pulmonary lesions in series of follow-up CT examinations despite administration of antibiotics and avid uptake of ^18^FDG on the PET scan. All cases were reviewed by our multidisciplinary lung tumor board to evaluate the operability, comorbidities, and performance status to facilitate a consensus of SBRT treatment. Each patient signed the written consent form for radiation therapy before the implementation of SBRT treatment. This retrospective study was approved by the institutional review board, thus the requirement for informed consent was waived.

### SBRT Implementation

A combination of vacuum body cushion and thermoplastic body mask was utilized for position immobilization. Patients were coached to lay comfortably on a customized vacuum body cushion with both arms extending overhead with hands gripping the position bar. The vacuum cushion provided support and immobilization from the bottom and sides. In the meanwhile, the thermoplastic body mask applied pressure from the anterior direction to strengthen immobilization and inhibit the respiratory amplitude. Abdominal compression was applied to patients with tumor motion exceeding 15 mm in the longitudinal direction according to RTOG 3502 protocol.

CT simulation was conducted using a three-phase scanning protocol (free breathing, end of expiration, and end of inspiration) or 4D-CT acquisition protocol (Siemens Somatom Sensation; Siemens Healthineers Corporation, Germany). Afterwards, reconstruction images of 2-mm slice thickness were transferred to TPS workstation (Eclipse 8.5; Varian Co.) for contouring and treatment planning. Gross tumor volume (GTV) was contoured on each three-phase CT imaging series under lung window setting with no expansion, then combined to produce the ITV projected to free-breathing CT series. For the 4D-CT scans, the ITVs were derived from the summed GTVs from all respiratory phases or, alternatively, directly contoured on the maximum intensity projection CT dataset. The PTV was created by expanding the ITV with margins of 3 mm in posteroanterior/lateral planes and 5 mm in the craniocaudal plane to compensate for daily motion variations as well as set-up errors (2, 11–13).

Structures and organs at risk, including lungs, trachea, main bronchus, ribs, esophagus, heart, spinal cord, and brachial plexus (in cases with tumors located in the lung apex), were delineated on the reference images. Based on patients' physical condition and tumor location, individualized dose prescription was administrated with ≥90% of PTV being encompassed by ≥90% of the prescription dose. Besides, SBRT plans were evaluated according to RTOG 0813 using parameters such as the ratio of prescription isodose volume to the PTV volume (R100%), the ratio of 50% prescription isodose volume to the PTV volume (R50%), and maximum dose at 2 cm from PTV in any direction (D2cm) (14). Each SBRT plan should respect all critical organ dose-volume constraints: for lung, V20 <10%, V12.5 <15%; for esophagus, maximum dose <35 Gy at 0.1 cc; for trachea and main bronchus, no hot point (0.1 cc) over 32 Gy; for ribs in the fields, V30 <30 cc; for spinal cord, maximum dose <32 Gy at 0.1 cc.

All patients received treatment on a linear accelerator (Trilogy; Varian Medical Systems, Inc.). Treatment course was completed within 2 weeks. For plans with four or five fractions, treatment was delivered every other day; for those with more fractions, consecutive treatment was applied. Each treatment was image guided with cone-beam CT, including pre-treatment, intra-fractional, and post-treatment. All imaging was online reviewed by attending physicians and medical physicists to validate tumor position and correct errors.

### Follow-Up and Assessment of SRP

Patients were followed up routinely every 3 months in the first year. Tumor measurements at each follow-up were carried out using the Response Evaluation Criteria in Solid Tumors (RECIST) version 1.0, in which local control failure is defined as at least a 20% increase in the longest diameter relative to the previous baseline smallest longest diameter. As the most common radiation-related toxicity, the diagnosis of RP was based on the clinical symptoms and radiographic findings on consecutive follow-up CT imaging. According to NCI Common Terminology Criteria for Common Adverse Events 5.0 (CTCAE 5.0), RP is classified into five grades by severity: grade 1 RP is non-symptomatic or mild symptomatic without medication requirement; grade 2 RP is symptomatic and required medical intervention, but did not interfere with daily activities; grade 3 RP requires hospitalization and intravenous hormonal treatment and oxygen; grade 4 RP needs mechanical ventilation; grade 5 RP is irreversible fatal pneumonitis. The primary endpoint of this study was the incidence of RP events grades 2–5, which was defined as SRP. Time to SRP was recorded since the start of radiation therapy, with disease recurrence or death considered censoring events.

### Statistical Analysis

As hypothesized, a BED-based uniform dose constraint to normal lung tissue would be more applicable in treatment planning evaluation and protocol compliance in cases with variable SBRT fractionations. To calculate the biological effective dose received by normal lung tissue, we converted actual physical dose to BED using the following formula derived from the linear-quadratic (L-Q) model: BED (Gy) = n^*^d^*^ [1 + *d*/(α/β)], where *n* and *d* are the number and size of the dose fractions, and the ratio of α/β is assumed as 3 and 10 Gy for normal lung tissue and tumor, respectively (15). In the present study, MLD, tumor BED, lung V_BED5_, and lung V_BED10_ through V_BED200_ with an increment of 10 Gy were selected as the dosimetric factors to be analyzed. V_BEDx_ means the percentage of normal lung volume receiving dose over *x* Gy of BED.

Besides, parameters including gender, age, Eastern Cooperative Oncology Group performance status (ECOG PS), the presence of chronic obstructive pulmonary disease, chemotherapy history before radiotherapy, tumor diameter, location and histology, and the sizes of GTV and PTV were factored into the occurrence and severity of SRP.

Fisher's exact test and the independent *t*-test were used for univariate analysis of the association of dosimetric and clinical factors with the occurrence of SRP. Multivariate logistic regression analysis was conducted to investigate the significant factors (*p* < 0.05) obtained from the first step to assess their relative important association with SRP. Kaplan–Meier estimates of cumulative incidence of SRP over time were generated with log-rank tests used across different groups. Then the receiver operating characteristic (ROC) curve was generated to compare the performance of V_BEDx_ and traditional V5, V20 of non-target lung for predicting risk of SRP and obtain the most optimal cut-off value. We further used each cut-off value of V_BEDx_ parameters that was identified from the ROC curve to generate a biologically effective dose-volume histogram (BEDVH) of normal lung tissue to reduce the risk of SRP. All statistical tests were processed using the Statistical Package of Social Sciences (SPSS version 19.0, Chicago, IL) and two-sided with *p* ≤ 0.05 indicative of statistical significance.

## Results

**Figure 1:**
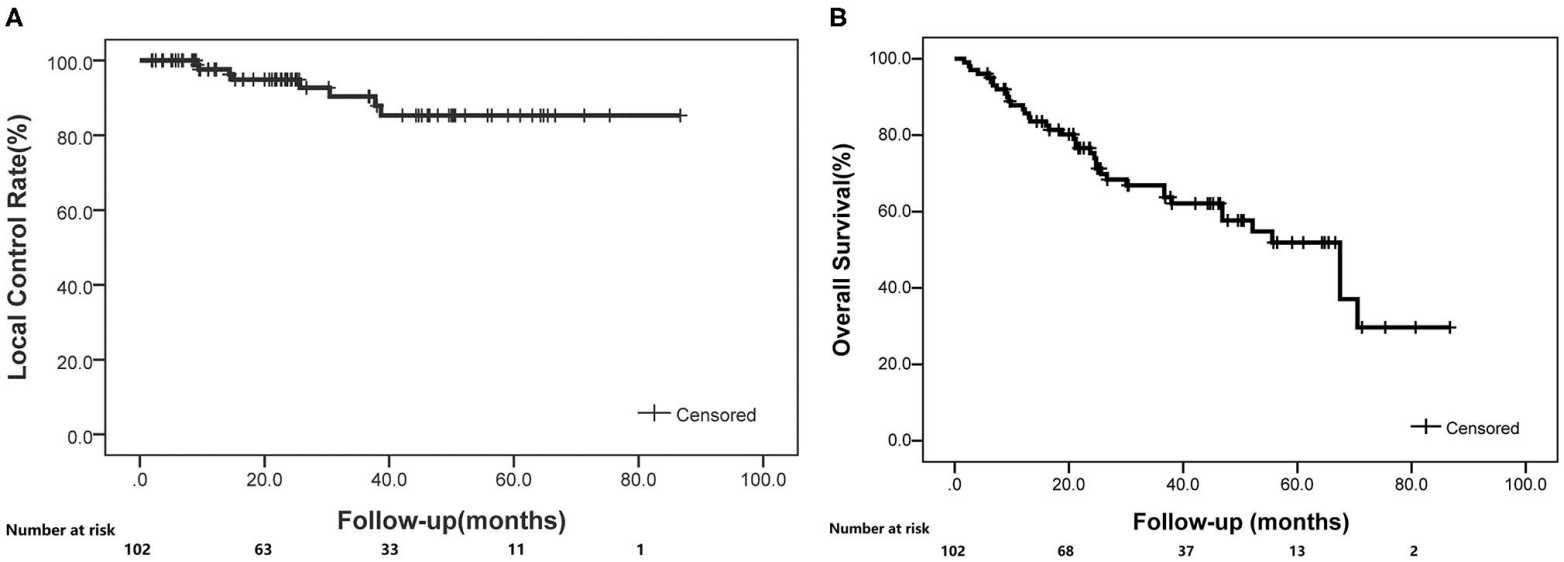
The local control and overall survival rates for enrolled patients treated with SBRT (K-M curve). **(A)** Local control rates through follow-up. **(B)** Overall survival rates through follow-up.

A total of 109 patients met the initial inclusion criteria, and 7 patients were lost to follow-up, leaving 102 patients as the subjects of this analysis. Of those, the median age was 77 years (range, 39–94 years). Sixty-eight (67%) patients were males and 44 (33%) patients were females. All patients completed the radiotherapy as planned with a prescription dose of 48–75 Gy in 4–12 fractions. The mean BED_10_ (with the α/β ratio of 10 Gy) estimated for the targeted tumor was 95.8 ± 11.4 Gy, and BED_3_ (with the α/β ratio of 3 Gy) was 186 ± 30.1 Gy for the non-targeted normal lung as shown in Table 1. The median follow-up period was 25.4 months (range, 1.7–86.7 months). At 1, 3, and 5 years, the local control rates were 97.6, 90.4, and 85.3% (Figure 1A), whereas the overall survival rates were 86.8, 66.9, and 51.9%, respectively (Figure 1B).

**Table 1:** Treatment and characteristics of patients with lung malignant tumors treated by SBRT.

**Characteristic**	**Value**	**%**
**Gender**		
Male (*n*)	68	66.7
Female (*n*)	34	33.3
**Age**		
Median (range)	77 (39-94)	/
**Tumor location**		
RUL (*n*)	30	29.4
RML (*n*)	13	12.8
RLL (*n*)	14	13.7
LUL (*n*)	36	35.3
LLL (*n*)	9	8.8
**Histology**		
Primary (*n*)	72	70.6
Metastatic (*n*)	30	29.4
**ECOG PS**		
0	20	19.6
1	55	53.9
2	27	26.5
**Diameter (mm)**		
Mean ± SD	26.1 ± 12.0	/
**GTV (cm**^**3**^**)**		
Mean ± SD	11.8 ± 17.3	/
**PTV (cm**^**3**^**)**		
Mean ± SD	25.6 ± 21.9	/
**Radiation schedules**		
5 Gy × 10–12Fx	17	16.7
6 Gy × 8–10Fx	38	37.3
7.5 Gy × 8–10Fx	34	33.3
8–12 Gy × 4–8Fx	13	12.7
**Chemotherapy**	20	19.6
Platinum	17	77.3
Pemetrexed	15	68.2
Others	7	31.8
**COPD (*****n*****)**	20	19.6
**BED (Gy)**		
Mean ± SD	95.8 ± 11.4	/
**Follow-up (months)**		/
Median (range)	25.4 (1.7–86.7)	/

*SBRT, stereotactic body radiation therapy; RUL, right upper lobe; RML, right middle lobe; RLL, right lower lobe; LUL, left upper lobe; LLL, left lower lobe; ECOG PS, Eastern Cooperative Oncology Group performance status; GTV, gross tumor volume; PTV, planning target volume; COPD, chronic obstructive pulmonary disease; BED, biologically effective dose*.

During the follow-up, 11 (10.8%) patients developed SRP: 9 (8.8%) grade 2 and 2 (2.0%) grade 3. No grade 4 or 5 RP was observed. The median interval from the completion of radiotherapy to the development of SRP was 3.4 months (range, 1.1–4.4 months). Only one patient suffered rib fracture 14 months after radiotherapy. Other adverse effects such as acute esophagitis, brachial plexopathy, and cardiac toxicity were not recorded.

The univariate analysis showed that none of the baseline clinical characteristics, including gender, age, chronic obstructive pulmonary disease, ECOG PS, chemotherapy history, tumor location, tumor histology, diameter, and GTV or PTV volume, had statistical significance with the risk of SRP (Table 2). All dosimetric factors (MLD, lung V_BED5−170_, and V5, V20) except tumor BED and V_BED180−200_ were correlated with SRP (*p* < 0.01) and thus were included in the multivariate analysis.

**Table 2:** Univariate and multivariate analysis of factors predicting SRP.

	**No-SRP group**	**SRP group**	***P* univariable**	***P* multivariable**
Gender			0.173	/
Male	63	5		
Female	28	6		
Tumor location			0.947	/
RUL	26	4		
RML	12	1		
RLL	12	2		
LUL	33	3		
LLL	8	1		
Chemotherapy			0.219	/
Yes	16	4		
No	75	7		
COPD			0.219	/
Yes	16	4		
No	75	7		
Histology			0.418	/
Primary (*n*)	62	10		
Metastatic (*n*)	29	1		
ECOG PS			0.633	/
0	19	1		
1	48	7		
2	24	3		
Age (years)	76 (41–94)	76 (39–91)	0.888	/
MLD (Gy)	291.12 ± 122.74	428.55 ± 109.95	0.002	0.884
Diameter (mm)	26.05 ± 12.05	26.60 ± 12.47	0.891	/
GTV (cm^3^)	11.56 ± 17.71	13.64 ± 13.69	0.709	/
PTV (cm^3^)	25.31 ± 22.97	27.94 ± 10.15	0.506	/
BED (Gy)	95.63 ± 11.62	96.75 ± 9.22	0.717	/
Lung V_BED5_ (%)	16.55 ± 7.85	25.23 ± 7.85	0.001	0.715
Lung V_BED10_ (%)	9.85 ± 4.64	17.30 ± 5.82	0.002	0.486
Lung V_BED20_ (%)	6.05 ± 3.21	11.21 ± 3.55	<0.001	0.653
Lung V_BED30_ (%)	4.16 ± 2.37	8.52 ± 2.81	<0.001	0.787
Lung V_BED40_ (%)	3.01 ± 1.76	6.82 ± 2.30	<0.001	0.383
Lung V_BED50_ (%)	2.29 ± 1.38	5.42 ± 2.01	<0.001	0.469
Lung V_BED60_ (%)	1.79 ± 1.12	4.43 ± 1.78	0.001	0.337
Lung V_BED70_ (%)	1.44 ± 0.93	3.66 ± 1.59	0.001	<0.001
Lung V_BED80_ (%)	1.17 ± 0.78	3.06 ± 1.41	0.001	0.329
Lung V_BED90_ (%)	0.96 ± 0.66	2.58 ± 1.26	0.002	0.401
Lung V_BED100_ (%)	0.78 ± 0.57	2.18 ± 1.14	0.002	0.477
Lung V_BED110_ (%)	0.64 ± 0.49	1.84 ± 1.03	0.003	0.512
Lung V_BED120_ (%)	0.52 ± 0.43	1.55 ± 0.96	0.005	0.560
Lung V_BED140_ (%)	0.33 ± 0.32	1.06 ± 0.86	0.018	0.549
Lung V_BED150_ (%)	0.26 ± 0.27	0.89 ± 0.79	0.024	0.629
Lung V_BED160_ (%)	0.19 ± 0.23	0.74 ± 0.71	0.028	0.827
Lung V_BED170_ (%)	0.14 ± 0.19	0.61 ± 0.64	0.036	0.935
Lung V_BED180_ (%)	0.10 ± 0.16	0.49 ± 0.58	0.051	/
Lung V_BED190_ (%)	0.07 ± 0.13	0.38 ± 0.53	0.082	/
Lung V_BED200_ (%)	0.04 ± 0.10	0.29 ± 0.49	0.125	/
Lung V_5_ (%)	14.48 ± 7.08	22.19 ± 7.10	0.005	0.847
Lung V_20_ (%)	3.51 ± 2.10	6.78 ± 2.07	<0.001	0.636

*SRP, symptomatic radiation pneumonitis; RUL, right upper lobe; RML, right middle lobe; RLL, right lower lobe; LUL, left upper lobe; LLL, left lower lobe; COPD, chronic obstructive pulmonary disease; ECOG PS, Eastern Cooperative Oncology Group performance status; MLD, mean lung dose; GTV, gross tumor volume; PTV, planning target volume; BED, biologically effective dose*.

**Figure 2:**
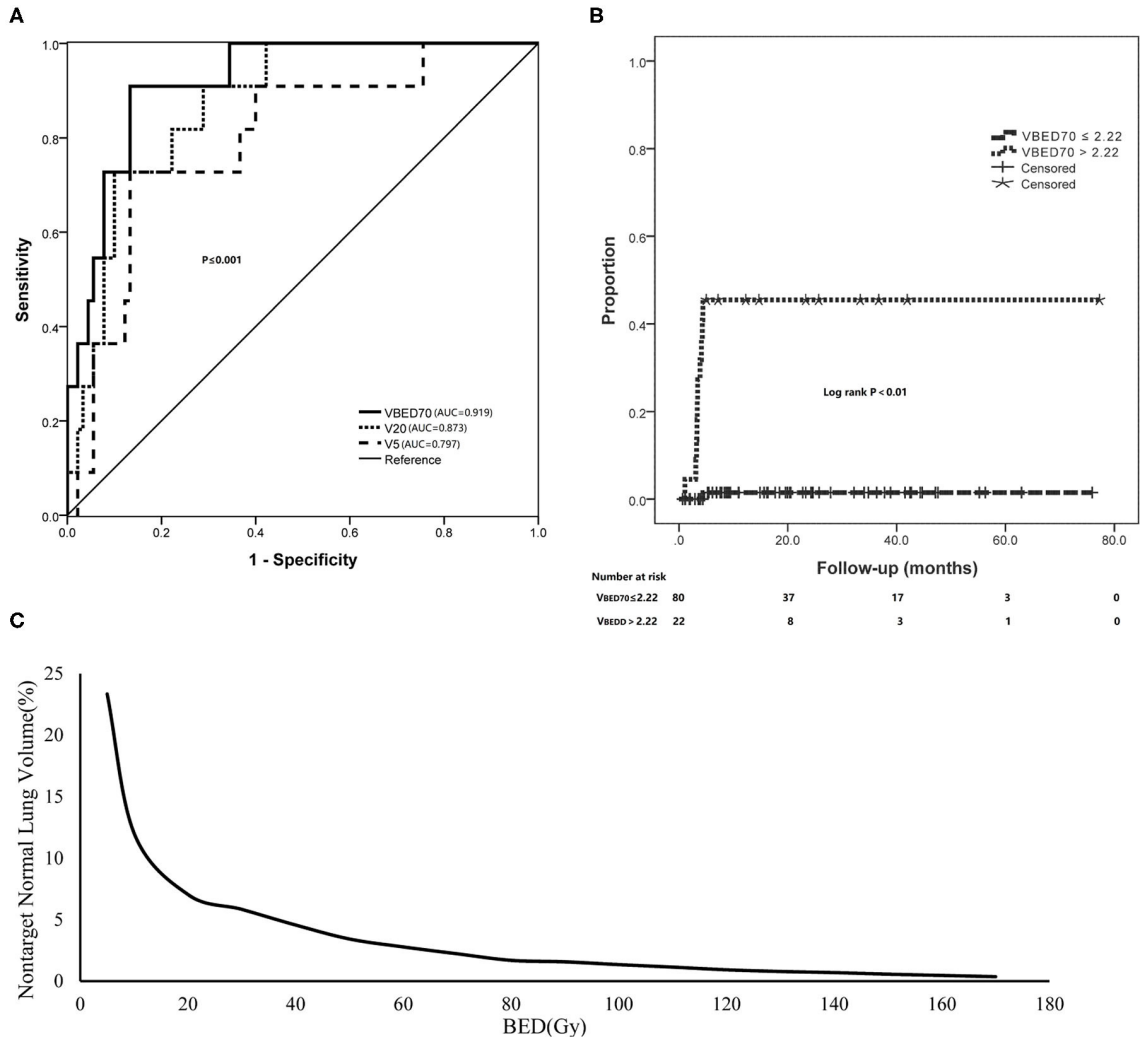
The adaption of BED of non-target lung volume for predicting SRP. **(A)** The ROC curve analysis of V_BED70_ in comparison with V_5_ and V_20_. **(B)** Using V_BED70_ (2.22%) as the dose constraint threshold dramatically reduced the incidence of SRP. **(C)** A sample BED-volume histogram (BEDVH) for non-target normal lung showed the tolerable and intolerable volumes corresponding to indicated BEDs.

**Figure 3:**
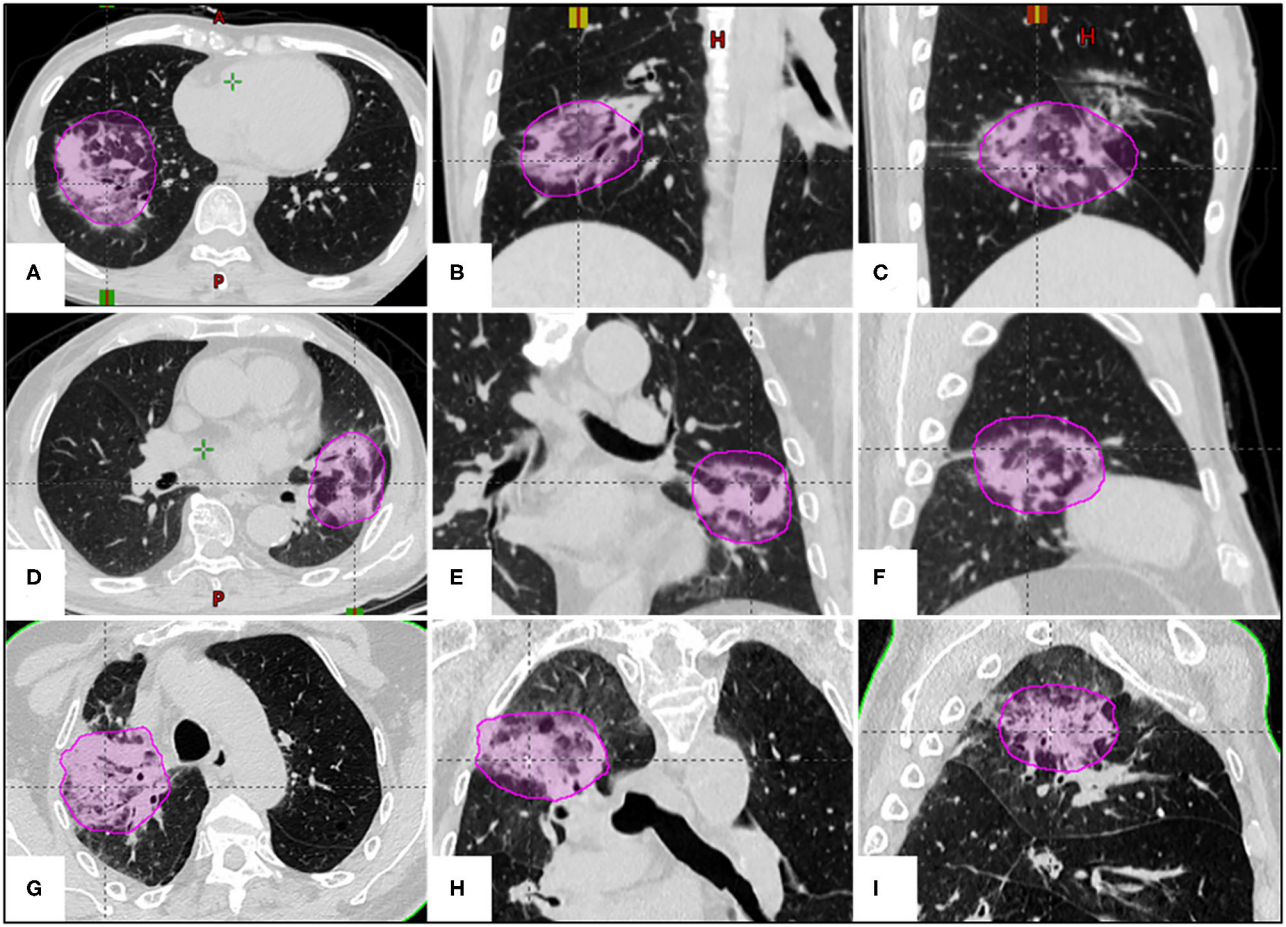
The examples of correlation between lung BED70 (magenta circle) and SRP in three different SBRT fractionations. **(A–C)** A 39-year-old female patient with adenocarcinoma in the right lower lobe received a prescription dose of 50 Gy in 5 fractions (V_BED70_ = 4.88%); **(D–F)** a 76-year-old male patient with metastatic lung tumor in the left upper lobe received a prescription dose of 60 Gy in 8 fractions (V_BED70_ = 3.78%); **(G–I)** an 85-year-old male patient with squamous cell carcinoma in the right upper lobe received a prescription dose of 60 Gy in 10 fractions (V_BED70_ = 3.15%).

Subsequent multivariate analysis showed that the significant factor was V_BED70_ (*p* < 0.001). Further analysis using ROC curve for V_BED70_ demonstrated that the area under the curve (AUC) was 0.919 and the optimal cut-off value was 2.22%, with a sensitivity of 0.91 and a specificity of 0.87. Comparing with conventional V5 and V20, V_BED70_ has a better predictive performance with the AUC value of 0.797, 0.873, and 0.919, respectively (*p* = 0.001, *p* < 0.001, *p* < 0.001; Figures 2A, 3). Using V_BED70_ = 2.22% as the stratification factor, the cumulative incidence of SRP was 1.5% in patients with V_BED70_ ≤ 2.22% and dramatically rose to 45.5% in patients with V_BED70_ > 2.22% (*p* < 0.001; Figure 2B).

Furthermore, considering that dosimetric variables were correlated with each other, we used ROC analysis to establish the best cut-off values and assess the predictability of each parameter from V_BED5_ to V_BED170_. The incidence of SRP was significantly lower in the patients with cut-off values of V_BED5_ < 23.34%, V_BED10_ < 12.03%, V_BED20_ < 7.04%, V_BED30_ < 5.84%, V_BED40_ < 4.57%, V_BED50_ < 3.42%, V_BED60_ < 2.78%, V_BED70_ < 2.22%, V_BED80_ < 1.69%, V_BED90_ < 1.57%, V_BED100_ < 1.35%, V_BED110_ < 1.15%, V_BED120_ < 0.93%, V_BED130_ < 0.79%, V_BED140_ < 0.70%, V_BED150_ < 0.57%, V_BED160_ < 0.47%, and V_BED170_ < 0.36%, respectively (all *p* < 0.001). Finally, we used each optimal cut-off value of lung V_BED5−170_ parameters that was identified from the ROC curve to draw a BEDVH to reduce risk of SRP (Figure 2C).

## Discussion

Hypofractionated SBRT has dramatically improved the management of inoperable early-stage NSCLC and oligometastatic pulmonary diseases with excellent tumor control and overall survival in comparison with conventional radiotherapy (3, 16). In contrast to conventional radiotherapy routinely used for locally advanced NSCLC in combination with chemotherapy, SBRT delivers highly focused, high fractionated dose to a limited target volume, typically smaller than 5 cm in diameter with the mediastinal structures spared. Consequently, the irradiated lung volume in SBRT is much smaller than in conventional radiotherapy. As a result, the pulmonary toxicity profile featured by radiation pneumonitis differed between these two radiation treatment regimens. SBRT-related radiation pneumonitis, if occurred, is commonly characterized by few clinical symptoms, self-limiting, and focal radiological findings (consolidation, for instance) (17). However, severe pulmonary toxicity especially the SRP has been reported to be a potential risk for pulmonary fibrosis and functional insufficiency, which could exacerbate pre-existing compromised pulmonary function, leading to treatment failure and even death (18). Considering that a large majority of patients receiving SBRT have severe comorbidities or have been in fragile conditions, radiation pneumonitis, particularly SRP, should be prevented and/or actively managed.

Apparently, RP results from the direct exposure of lung tissue to radiation. The radiation dose and irradiated volume bear the most significant weight in the occurrence of RP. Prescription dose (or tumor BED) has been reported to be associated with tumor control and SRP incidence. Considering that the majority of our patients were fragile with limited pulmonary functional reserve, a moderate BED of 95.8 ± 11.4 Gy was applied which partly accounted for the relatively low incidence of SRP and the lack of correlation between tumor BED and SRP. In our study, the incidence of grade 2 and grade 3 RP was 8.8 and 2.0%, respectively, comparable with 11% reported in previous studies (9, 19). The percentage of non-target lung volume receiving more than a specific radiation dose (Vx) and mean lung dose (MLD) have been widely investigated for the correlation to SRP (20, 21). However, the results seem inconclusive. In a study conducted by Barriger et al., MLD and V20 were deemed as the significant risk factors of SRP (22). Contrarily, Matsuo et al. concluded that only PTV, V20, and V25 were RP indicators, whereas factors such as MLD, V5, V10, V15, V30, and V40 were not associated with RP (8). As both studies addressed regarding the limitation, variation of dose fractionation and biological effective dose may be the major confounding factor of inconsistent reported results. Despite that, V20 <10% has been recommended as the uniform dose constraint for normal lung in UK consensus on normal tissue dose constraints for SBRT regardless of 3, 5, or 8 fraction schedules (10).

In light of the aforementioned controversy, in this study we attempted to apply the concept of BED to unify the lung dose (lung BED) in variable SBRT fractionations and investigate the correlation between lung BED and SRP with an aim to establish a lung BED–based constraint to minimize the risk of SRP.

In the univariate analysis, we found that derived BED dosimetrics (dose-volume variables and MLD) were associated with SRP in patients receiving SBRT. In contrast, there was no significant difference in clinical variables between the SRP group and the non-SRP group. However, the multivariate analysis demonstrated that lung V_BED70_ was the only risk factor for SRP, whereas the risk factors for SRP reported in previous literature, such as V5, V20, and MLD, were not statistically different between patients with SRP and SRP-free patients in this study.

Furthermore, in the ROC analysis, V_BED70_ had better predictive performance with higher AUC value than V5 and V20. In the present study, V_BED70_ could predict SRP among patients treated with SBRT with rates of 45.5% for patients with V_BED70_ > 2.22 vs. 1.5% for those with V_BED70_ ≤ 2.22% (*p* < 0.001). Obviously, our study suggested that the lung BED volume parameter had reliable predictive value for severe SRP across various SBRT fractionation regimens.

Interestingly, the V_BED70_ of the two patients with RP grade 3 were 5.28 and 2.99%, which were not the highest in the SRP group. Because all the parameters from V_BED5_ to V_BED170_ were significant in the univariate analysis, we selected each corresponding optimal cut-off value obtained from the ROC to draw a tolerance curve based on lung V_BED_ (BEDVH), which may be more appropriate than single-point dosimetric constraints to optimize the SBRT planning.

Various clinical factors have been reported to be associated with SBRT-related SRP, including patient age, smoking status, performance status, and tumor location, diameter as well as concurrent chemotherapy (23). These factors should be taken into consideration with dosimetric parameters in evaluation of potential risk of SRP. For patients with mild symptoms, clinical observation with short-term prophylactic use of oral corticosteroid should be adequate. However, for patients with severe symptoms, hospitalization and systemic therapies including oxygen, glucocorticoids, and anti-inflammatory agents should be given to avoid symptomatic deterioration.

Two major limitations in this study should be addressed: the small sample size and relatively consistent tumor BED (95.8 ± 11.4 Gy). The former might create deviation in the SRP group and the non-SRP group, whereas the latter may only represent certain actual SBRT prescription as for some tumors a higher BED may be prescribed in many institutions. Hence, with the retrospective nature, the extrapolation of current results should be cautious before further prospective validation study is available. However, the most important point revealed from this study is that we should pay more attention to the application of the lung-BED concept in predicting SRP across various SBRT fractionation regimens. The other issue worthwhile to be mentioned is patient selection. According to our SBRT protocol, lung SBRT is applied to lesions with predominance of solid component and those ground-glass nodules (GGNs), either highly suspicious or diagnostic of lung cancer, are excluded, implying that current results may not be applicable to patients with GGNs treated with SBRT. Patients with GGNs are prone to develop SRP with increasing likelihood, severity, and uncertainties in extent after SBRT treatment according to our experience and limited reports (24, 25).

In conclusion, this retrospective study shows that the severity of SRP in SBRT tends to be highly correlated to the moderate or high dose received by lung tissues surrounding the target, which emphasizes the importance of restraining dose spillage in SBRT treatment planning. Also, lung BED may better predict SRP in patients receiving SBRT treatment with varying fractionations. Similar to tumor BED, the adoption of lung BED could facilitate treatment plan evaluation and comparison among patients as well as SBRT regimens. To limit the V_BED70_ below 2.22% by improving treatment plan quality may be favorable to reduce the incidence of SRP, which warranted further prospective validation in a large cohort of patients.

## Data Availability Statement

The raw data supporting the conclusions of this article will be made available by the authors, without undue reservation.

## Ethics Statement

The studies involving human participants were reviewed and approved by the Ethics Committee of Clinical Research of Fudan University Huadong Hospital. Written informed consent from the patients/participants was not required to participate in this study in accordance with the national legislation and the institutional requirements.

## Author Contributions

XZ proposed the conception and design of the study. YJ, YR, and XZ provided study materials of the patients. YJ, YR, WG, and LZ collected the assembly of data. YJ, YR, and XZ analyzed and interpreted the data. All authors edited and approved the final manuscript. All authors contributed to the article and approved the submitted version.

## Conflict of Interest

The authors declare that the research was conducted in the absence of any commercial or financial relationships that could be construed as a potential conflict of interest.

## References

[1] Chang JY Senan S Paul MA Mehran RJ Louie AV Balter P Stereotactic ablative radiotherapy versus lobectomy for operable stage I non-small-cell lung cancer: a pooled analysis of two randomised trials. Lancet Oncol. (2015) 16:630–7. 10.1016/S1470-2045(15)70168-3 25981812 PMC4489408

[2] Ball D Mai GT Vinod S Babington S Ruben J Kron T. Stereotactic ablative radiotherapy versus standard radiotherapy in stage 1 non-small-cell lung cancer (TROG 09.02 CHISEL): a phase 3, open-label, randomised controlled trial. Lancet Oncol. (2019) 20:494–503. 10.1016/S1470-2045(18)30896-9 30770291

[3] Otake S Goto T. Stereotactic radiotherapy for oligometastasis. Cancers. (2019) 11:133. 10.3390/cancers11020133 30678111 PMC6407034

[4] Bongers EM Botticella A Palma DA Haasbeek CJ Warner A Verbakel WF Predictive parameters of symptomatic radiation pneumonitis following stereotactic or hypofractionated radiotherapy delivered using volumetric modulated arcs. Radiother Oncol. (2013) 109:95–9. 10.1016/j.radonc.2013.10.011 24183862

[5] Lu C Lei Z Wu H Lu H. Evaluating risk factors of radiation pneumonitis after stereotactic body radiation therapy in lung tumor: meta-analysis of 9 observational studies. PLoS ONE. (2018) 13:e0208637. 10.1371/journal.pone.0208637 30521600 PMC6283643

[6] Boonyawan K Gomez DR Komaki R Xu Y Nantavithya C Allen PK. Clinical and dosimetric factors predicting grade ≥ 2 radiation pneumonitis after postoperative radiotherapy for patients with non-small cell lung carcinoma. Int J Radiat Oncol Biol Phys. (2018) 101:919–26. 10.1016/j.ijrobp.2018.04.012 29976504

[7] Ricardi U Badellino S Filippi AR. Stereotactic body radiotherapy for early stage lung cancer: history and updated role. Lung Cancer. (2015) 90:388–96. 10.1016/j.lungcan.2015.10.016 26791797

[8] Matsuo Y Shibuya K Nakamura M Narabayashi M Sakanaka K UekiN. Dose–volume metrics associated with radiation pneumonitis after stereotactic body radiation therapy for lung cancer. Int J Radiat Oncol Biol Phys. (2012) 83:e545–9. 10.1016/j.ijrobp.2012.01.018 22436782

[9] Baker R Han G Sarangkasiri S DeMarco M Turke C Stevens CW. Clinical and dosimetric predictors of radiation pneumonitis in a large series of patients treated with stereotactic body radiation therapy to the lung. Int J Radiat Oncol Biol Phys. (2013) 85:190–5. 10.1016/j.ijrobp.2012.03.041 22929858

[10] Hanna GG Murray L Patel R Harrow S Landau D Ahmed M UK consensus on normal tissue dose constraints for stereotactic radiotherapy. Clin Oncol. (2018) 30:5–14. 10.1016/j.clon.2017.09.007 29033164

[11] Yamazaki R Onimaru R Katoh N Inoue T Nishioka I Shirato H. Influence of respiration on dose calculation in stereotactic body radiotherapy of the lung. Radio Phys Technol. (2014) 7:284–9. 10.1007/s12194-014-0263-4 24643842

[12] Lin G Xiao H Zeng Z Xu Z He J Sun T. Constraints for symptomatic radiation pneumonitis of helical tomotherapy hypofractionated simultaneous multitarget radiotherapy for pulmonary metastasis from hepatocellular carcinoma. Radiother Oncol. (2017) 123:246–50. 10.1016/j.radonc.2017.02.015 28314468

[13] Hurkmans CW van Lieshout M Schuring D van Heumen MJ Cuijpers JP Lagerwaard FJ. Quality assurance of 4D-CT scan techniques in multicenter phase III trial of surgery versus stereotactic radiotherapy (radiosurgery or surgery for operable early stage (stage 1A) non-small-cell lung cancer [ROSEL] study). Int J Radiat Oncol Biol Phys. (2011) 80:918–27. 10.1016/j.ijrobp.2010.08.017 20950961

[14] Bezjak A Paulus R Gaspar LE Timmerman RD Straube WL Ryan WF. Safety and efficacy of a five-fraction stereotactic body radiotherapy schedule for centrally located non-small-cell lung cancer: NRG oncology/RTOG 0813 trial. J Clin Oncol. (2019) 37:1316–25 10.1200/JCO.18.00622 30943123 PMC6524984

[15] Fowler JF. The linear-quadratic formula and progress in fractionated radiotherapy. Br J Radiol. (1989) 62:679–94. 10.1259/0007-1285-62-740-679 2670032

[16] Palma D Visser O Lagerwaard FJ Belderbos J Slotman BJ Senan S. Impact of introducing stereotactic lung radiotherapy for elderly patients with stage I non-small-cell lung cancer: a population-based time-trend analysis. J Clin Oncol. (2010) 28:5153–9. 10.1200/JCO.2010.30.0731 21041709

[17] Linda A Trovo M Bradley JD. Radiation injury of the lung after stereotactic body radiation therapy (SBRT) for lung cancer: a timeline and pattern of CT changes. Eur J Radiol. (2011) 79:147–54. 10.1016/j.ejrad.2009.10.029 19954913

[18] Bütof R Kirchner K Appold S Löck S Rolle A Höffken G. Potential clinical predictors of outcome after postoperative radiotherapy of non-small cell lung cancer. Strahlenther Onkol. (2014) 190:263–9. 10.1007/s00066-013-0501-4 24413893

[19] Yamashita H Nakagawa K Nakamura N Koyanagi H Tago M Igaki H. Exceptionally high incidence of symptomatic grade 2-5 radiation pneumonitis after stereotactic radiation therapy for lung tumors. Radiat Oncol. (2007) 2:21. 10.1186/1748-717X-2-21 17553175 PMC1894806

[20] Hernando ML Marks LB Bentel GC Zhou SM Hollis D Das SK. Radiation-induced pulmonary toxicity: a dose-volume histogram analysis in 201 patients with lung cancer. Int J Radiat Oncol Biol Phys. (2001) 51:650–9. 10.1016/S0360-3016(01)01685-6 11597805

[21] Kwa SL Lebesque JV Theuws JC Marks LB Munley MT Bentel G. Radiation pneumonitis as a function of mean lung dose: an analysis of pooled data of 540 patients. Int J Radiat Oncol Biol Phys. (1998) 42:1–9. 10.1016/S0360-3016(98)00196-5 9747813

[22] Barriger RB Forquer JA Brabham JG Andolino DL Shapiro RH Henderson MA. A dose-volume analysis of radiation pneumonitis in non-small cell lung cancer patients treated with stereotactic body radiation therapy. J Radiat Oncol Biol Phys. (2012) 82:457–62. 10.1016/j.ijrobp.2010.08.056 21035956

[23] Robnett TJ Machtay M Vines EF McKenna MG Algazy KM McKenna WG. Factors predicting severe radiation pneumonitis in patients receiving definitive chemoradiation for lung cancer. J Radiat Oncol Biol Phys. (2000) 48:89–94. 10.1016/S0360-3016(00)00648-9 10924976

[24] Badiyan SN Bierhals AJ Olsen JR Creach KM Garsa AA Dewees T. Stereotactic body radiation therapy for the treatment of early-stage minimally invasive adenocarcinoma or adenocarcinoma in situ (formerly bronchioloalveolar carcinoma): a patterns of failure analysis. Radiat Oncol. (2013) 8:4. 10.1186/1748-717X-8-4 23286648 PMC3552761

[25] Louie AV Senan S Dahele M Slotman BJ Verbakel WF. Stereotactic ablative radiation therapy for subcentimeter lung tumors: clinical, dosimetric, and image guidance considerations. Int J Radiat Oncol Biol Phys. (2014) 90:843–9. 10.1016/j.ijrobp.2014.06.064 25585783

